# Diminishing evidence for torsinA-positive neuronal inclusions in DYT1 dystonia

**DOI:** 10.1186/s40478-016-0362-z

**Published:** 2016-08-17

**Authors:** Drew Pratt, Karin Mente, Shervin Rahimpour, Nancy A. Edwards, Sule Tinaz, Brian D. Berman, Mark Hallett, Abhik Ray-Chaudhury

**Affiliations:** 1 Laboratory of Pathology, National Cancer Institute, National Institutes of Health, Bethesda, MD USA; 2 Human Motor Control Section, National Institute of Neurological Disorders and Stroke, National Institutes of Health, 10 Center Drive, Room 7D42, Bethesda, MD 20892 USA; 3 Department of Neurosurgery, Duke University Medical Center, Durham, NC USA; 4 Surgical Neurology Branch, National Institute of Neurological Disorders and Stroke, National Institutes of Health, Bethesda, MD USA; 5 Department of Neurology, Yale School of Medicine, New Haven, CT USA; 6 Department of Neurology, University of Colorado Anschutz Medical Campus, Denver, CO USA

**Keywords:** DYT1, Dystonia, TorsinA, Inclusions

DYT1 dystonia, an early onset generalized dystonia, also known as Oppenheim’s dystonia, is an inherited isolated dystonia characterized by progressive generalized muscle spasms and sustained postures leading to significant disability [1]. The disease is inherited in an autosomal dominant manner with incomplete penetrance (30–40 %) and typically presents in childhood [2]. Patients harbor a 3-bp (GAG) deletion in the coding region of the *TOR1A* gene on chromosome 9q34 that encodes the protein torsinA [3]. This deletion corresponds to loss of a single glutamate at amino acid residue 302 or 303 (torsinA ΔE) [4]. The function of wildtype torsinA has been speculative, but relatively recent studies have demonstrated its involvement in protein trafficking, quality control, secretion, and degradation (for review, see Dauer 2014 [5]). The pathogenic mechanism leading to disease as a result of this deletion is thought to likely involve disruption of sensorimotor circuit development and function [6]. Recent evidence also suggests striatal cholinergic dysfunction, or dysregulation, as a potential mechanism underlying the pathophysiology of DYT1 dystonia [7].

Various in vitro and transgenic animal models of DYT1 dystonia have demonstrated altered cell morphology and nuclear changes at the light microscopic level, including torsinA accumulation and torsinA-positive inclusions in the brainstem (see Dauer 2014 [5] and Oleas et al. 2013 [8] for review of cell culture and animal model findings, respectively). Contrary to findings in animal tissues, no *consistent* or specific histopathologic changes have been noted in postmortem neuropathologic studies of patients with DYT1 dystonia (Table 1). Subcellular changes (e.g., inclusions) in DYT1 brains have been described in a small human postmortem study [9], but their specificity remains controversial. That study reported the presence of perinuclear and intranuclear inclusion bodies and protein accumulation in neurons that were immunoreactive for torsinA and ubiquitin, and co-localized with choline acetyltransferase (ChAT) and a nuclear envelope marker, lamin A/C, in the midbrain and pontine reticular formation (pedunculopontine nucleus (PPN), cuneiform nucleus (CN), and periaqueductal grey (PAG)); these changes were notably absent in controls. A more recent study, however, failed to confirm the presence of immunoreactive inclusions in human DYT1 dystonia brain tissue (using antibodies to ubiquitin and p62) [10]. Additionally, two earlier studies failed to reveal inclusion bodies, aggregates, or aberrant staining for torsinA in DYT1 brains and in other dystonia (non-DYT1) cases [11, 12].

**Table 1:** DYT1 dystonia neuropathology studies

References	Subjects	Inclusions	Key antibodies	Subcellular TorsinA immunoreactivity
Walker et al. 2002 [11]	1 DYT1 Dystonia subject	No	Anti-torsinA (rabbit polyclonal: AA residues 323–332)	Nucleus, cytoplasm, and dendrites in both DYT1 dystonia and control groups
	4 control subjects	No		
Rostasy et al. 2003 [12]	5 DYT1 Dystonia subjects	No	Anti-torsinA (rabbit polyclonal TAB1: AA residues 299–312; mouse monoclonal D-MG10: AA residues 208–249)	Cytoplasm, dendrites, and axons in both DYT1 dystonia and control groups
	20 control subjects	No		
McNaught et al. 2004 [9]	4 DYT1 Dystonia subjects	Yes: midbrain, pons	Anti-torsinA (rabbit polyclonal; AA residues 323–332), Anti-ubiquitin, Anti-UPC, Anti-ChAT	Perinuclear and intranuclear inclusions in DYT1 dystonia group only
	4 control subjects	No		
Paudel et al. 2014 [10]	7 DYT1 Dystonia subjects	No	Anti-ubiquitin, Anti-p62	N/A

*AA* amino acid, *ChAT* choline acetyl transferase, *UPC* ubiquitin-protein conjugate

**Fig. 1:**
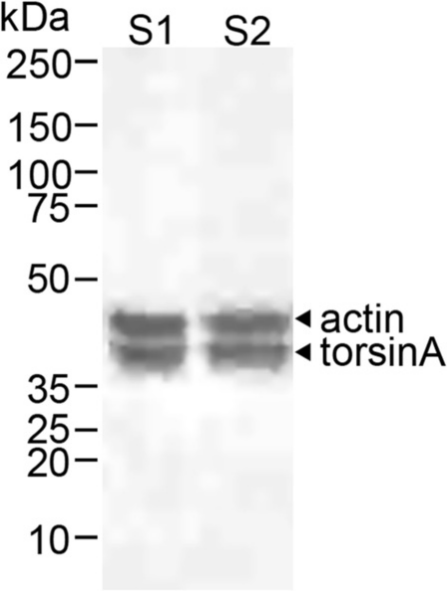
Specificity of the torsinA antibody MAB5550 by Western blot analysis. This Western blot of two control human brain homogenates (labeled as S1 and S2) was performed with anti-torsinA and anti-actin primary antibodies. The location and corresponding molecular weight for torsinA (37 kDa) is shown. Actin (42 kDa) was used as a loading control

Here, we sought to analyze genetically confirmed DYT1 brain specimens with antibodies to torsinA, ubiquitin protein conjugate (UPC), and ChAT in an attempt to identify the previously reported intracellular immunoreactive protein inclusions/aggregates in the midbrain as well as in other cholinergic nuclei, including the striatum. Our study included six brain samples from DYT1 patients (mean age 83.0 ± 9.1 years; all female), procured from the University of Maryland Brain and Tissue Bank (BTB); demographic and autopsy data from four of our DYT1 subjects matched those reported previously [10] and most likely represent tissue from the same subjects. In our study, three patients were clinically symptomatic and the remaining were non-manifesting carriers of the DYT1 mutation. Control brain tissue samples from seven subjects without clinical evidence of dystonia or other movement disorders were matched for age and sex (mean age 83.4 ± 8.0 years; all female). Formalin-fixed paraffin-embedded (FFPE) tissue sections from the striatum, motor and sensory cortices in all patients and controls, and from the brainstem (midbrain/pons, including PPN, CN, and PAG) in five of six DYT1 cases and all controls were evaluated. Routine hematoxylin-eosin staining was unrevealing. Immunohistochemistry (IHC) with antibodies directed to the following antigens were used: torsinA (Chemicon, MAB5550), ChAT (Millipore, AB144P), and ubiquitin protein conjugate (Millipore, 09–408). Additionally, IHC with anti-beta amyloid (Dako, M0872), anti-phospho-tau (AT8, Pierce, MN1020), and anti-alpha synuclein (Cell Signaling, 2647) antibodies along with Bielschowsky silver staining excluded significant neurodegenerative disease. Double staining IHC was performed for torsinA and ChAT using an automated immunostainer (Leica Bond-Max, Buffalo Grove, IL). Western blot analysis was performed with anti-torsinA on fresh-frozen control human brain tissue lysates and appropriate molecular weight labeling for the torsinA protein (37 kDa) was confirmed (Fig. 1). To further evaluate intracellular chromogen (3,3-diaminobenzidine and Fast Red) localization, multispectral imaging (CRi Nuance V.2.8, Woburn, MA) was employed on double-labeled fixed tissue stained with torsinA and ChAT.

**Fig. 2:**
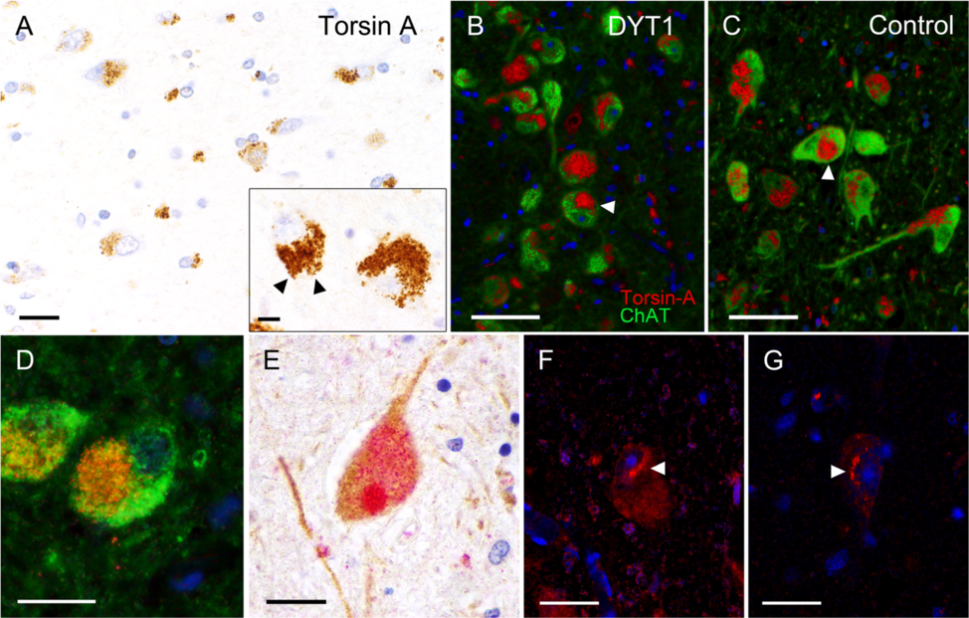
Cellular distribution of torsinA and ChAT in DYT1 and control striatum. **a** Normal staining pattern and cellular distribution of torsinA in the human striatum (scale bar, 20 μm). TorsinA shows granular cytoplasmic staining (inset, arrowheads; scale bar, 10 μm). **b**, **c**, **d** Fluorescence-like multispectral image (gated immunohistochemical chromogen signal) of torsinA protein (red) reveals circumscribed collections in the cytoplasm of both DYT1 and control neurons (arrowheads; scale bars, 20 μm). ChAT (green) shows diffuse immunoreactivity within the cytoplasm and processes in both DYT1 and controls. **e** This section of an unaffected control shows an inclusion-like aggregate immunoreactive to torsinA (red) (scale bar, 20 μm). **f**, **g** Also identified was perinuclear accumulation of torsinA (red) seen in DYT1 tissue, thought to be a response to cellular stress (scale bars, 20 μm)

No specific cytoplasmic, perinuclear, or intranuclear torsinA-immunoreactive inclusions were identified in our DYT1 tissues. Immunoreactivity for torsinA, ChAT, and UPC in both DYT1 and control groups yielded similar results and predominantly showed normal staining patterns for these endogenous cellular proteins. In both groups, torsinA demonstrated granular cytoplasmic staining in all regions evaluated, as well as punctate immunoreactivity in the neuropil (Fig. 2a). In addition to its widespread neuroanatomic distribution, cellular localization studies have shown that wildtype torsinA is limited to neuronal cytoplasm (endoplasmic reticulum), nuclear envelope, and processes. It should be noted that occasional variations in the staining pattern of torsinA were seen in both DYT1 and control tissues. Apparent cytoplasmic aggregation of the torsinA protein was seen in both groups, with these changes most prominent in large striatal cholinergic interneurons (Fig. 2b, c, d). Inclusion body-like immunoreactivity seen in a control subject (Fig. 2e) exemplifies the non-specific nature of these changes. Occasional neurons in the striatum in two DYT1 patients demonstrated perinuclear accumulation or accentuation of torsinA (Fig. 2f, g); this change was rare and not seen in control tissue. The staining patterns and distribution for ChAT and UPC were similar between both groups. ChAT showed diffuse expression in neuronal cell bodies and processes, while UPC demonstrated predominantly cytoplasmic immunoreactivity without evidence of inclusion bodies in the aforementioned brain regions.

Our results add to the growing body of evidence that there are no consistent torsinA immunoreactive protein inclusions associated with, or specific to, DYT1 dystonia in humans. In cell culture studies of neural cells transfected with mutant torsinA, cells demonstrated large perikaryal inclusions ultrastructurally composed of spheroid whorled membranes [13]. The location of the collections appeared to be mutation status-related, with overexpression of mutant torsinA forming inclusions adjacent to the nuclear membrane and overexpressed wildtype protein aggregating within the cytoplasm. This latter feature is in keeping with our findings but was nonspecific and may possibly represent a response of wildtype torsinA to cellular stress (e.g., agonal state). The perinuclear accentuation of torsinA immunoreactivity we observed has previously been described in cultured cells exposed to oxidative stress [14]. Lastly, it has been suggested that endogenous torsinA levels may be lower in vivo as compared to those in transfected cells due to overexpression in cell culture models, which may affect subcellular localization of torsinA [15].

Potential reasons for recurrent failure to demonstrate inclusions in human DYT1 tissue have been noted [5, 10, 12] and deserve mention here. Foremost, DYT1 dystonia is rare and tissue availability for sufficient sample size analysis is very limited; this is a limitation in the context of an incompletely penetrant disease, as disease-specific changes may be related to clinically apparent disease. Notably, while our evaluation was already limited to six DYT1 patients, only five included brainstem tissue (including the PPN, CN, and PAG) for evaluation. Second, studies involving human and animal tissues have failed to recapitulate the immunoreactive protein aggregates observed in culture cells overexpressing torsinA (see Dauer [5] for review), supporting the suspected lack of neurodegeneration in the disease. The human tissue studies that failed to identify torsinA inclusions used various anti-torsinA antibodies directed against different epitopes, which further supports that lack of inclusion body formation in DYT1 dystonia. Furthermore, there is strong evidence linking altered cholinergic striatal function, and its inputs from other structures such as the brainstem, cerebellum and thalamus, to the underlying pathophysiology in DYT1 dystonia (for review see Eskow Jaunarajs et al. [16]). Indeed, postmortem analyses of the putamen of DYT1 subjects have shown decreased levels of cholinergic markers [7].

In conclusion, there is little evidence supporting the presence of specific cellular morphologic changes in DYT1 dystonia at the light microscopic level in human tissue. The significance and specificity of the changes we observed are best addressed with larger postmortem studies combining histopathology and disease-specific cell models, such as patient-specific induced pluripotent stem cells (iPSCs). Use of iPSC-derived neurons would allow the subcellular localization of torsinA, and any inclusions, to be explored in living cells without altering the level of torsinA expression.
